# Sad faces increase the heartbeat-associated interoceptive information flow within the salience network: a MEG study

**DOI:** 10.1038/s41598-018-36498-7

**Published:** 2019-01-23

**Authors:** Jaejoong Kim, Hyeong-Dong Park, Ko Woon Kim, Dong Woo Shin, Sanghyun Lim, Hyukchan Kwon, Min-Young Kim, Kiwoong Kim, Bumseok Jeong

**Affiliations:** 1 Graduate School of Medical Science and Engineering, Korea Advanced Institute for Science and Technology (KAIST), 291 Daehak-ro, Yuseong-gu, Daejeon, 34141 Republic of Korea; 2 Laboratory of Cognitive Neuroscience, Center for Neuroprosthetics and Brain Mind Institute, Ecole Polytechnique Fédérale de Lausanne (EPFL), 9 Chemin des Mines, 1202 Geneva, Switzerland; 3 Department of Neurology, Chonbuk National University Hospital, Chonbuk National University Medical School, JeonJu, Korea; 4 Center for Biosignals, Korea Research Institute of Standards and Science, Daejeon, South Korea; 5 KI for Health Science and Technology, KAIST Institute, KAIST, 291 Daehak-ro, Yuseong-gu, Daejeon, 34141 Republic of Korea

## Abstract

The somatic marker hypothesis proposes that the cortical representation of visceral signals is a crucial component of emotional processing. No previous study has investigated the information flow among brain regions that process visceral information during emotional perception. In this magnetoencephalography study of 32 healthy subjects of either sex, heartbeat-evoked responses (HERs), which reflect the cortical processing of heartbeats, were modulated by the perception of a sad face. The modulation effect was localized to the prefrontal cortices, the globus pallidus, and an interoceptive network including the right anterior insula (RAI) and dorsal anterior cingulate cortex (RdACC). Importantly, our Granger causality analysis provides the first evidence for the increased flow of heartbeat information from the RAI to the RdACC during sad face perception. Moreover, using a surrogate R-peak analysis, we have shown that this HER modulation effect was time-locked to heartbeats. These findings advance the understanding of brain-body interactions during emotional processing.

## Introduction

According to the James-Lange theory and the somatic marker hypothesis, emotional feelings are the mental experience of bodily states^1,2^. Various feelings are hypothesized to subsequently emerge from the cortical representation of bodily sensation, which is called interoception^1^. Therefore, it is important to investigate interoceptive signal-induced cortical processing during emotional experiences. However, given that signals from internal organs cannot be identified without explicit measuring devices, such as electrocardiography (ECG), it is difficult to investigate the brain activity that is directly evoked by interoceptive signals. Heartbeat-evoked responses (HERs), which are obtained by averaging electrophysiological signals time-locked to heartbeats, provide one way to investigate the cortical processing of interoceptive signals. HERs are known to reflect the cortical processing of heartbeats and have been reported to be associated with heartbeat perception accuracy^3^, suppressed by pain perceptions^4^, and modulated by empathy feelings^5^. HER amplitudes are also attenuated in mood-related psychiatric disorders, including depression^6^ and borderline personality disorder^7^, suggesting a potential link between HERs and aberrant emotional processing. Moreover, several recent studies have successfully shown, using HERs, that cortical interoceptive processing is modulated by emotional processing. One study reported that HERs were modulated by emotional arousal^8^. Another study reported that HERs in infants were modulated by video clips showing fearful and angry facial expressions^9^. Finally, one study, using high-density electroencephalography (EEG) and natural affective scenes, localized the source of HERs to a frontal-insular-temporal network including the anterior insula (AI) and anterior cingulate cortex (ACC)^10^. The AI and ACC both have topological organized maps of the body^1^ and are co-activated in the majority of neuroimaging studies of emotion^11^. Therefore, these regions are hypothesized to conjointly act during emotional processing, with the AI acting as an input region that integrates interoceptive information and the ACC acting as an output region that uses integrated interoceptive information to generate emotion-related responses (the responses could be related to an autonomic/cognitive/behavioural domain)^12^. However, the modulation of the directional interoceptive information flows between these regions during emotional processing has not yet been investigated. Therefore, in this study, we aimed to identify the modulation of the directional interoceptive information flows during emotional perception using HERs. To this end, we used emotional faces and emotional emoticons that conveyed text-based emotions to evoke an emotional feeling while measuring the HERs with magnetoencephalography (MEG). We suspected that while emotional emoticons and emotional faces convey similar types of emotional information, their interoceptive processing could be different, as can be inferred from our previous fMRI study^13^. In that study, the activation of the insula was not found in the text-based emoticon condition, while the other activated regions overlapped with brain regions that are typically activated by emotional faces^13^. To verify the precise source of the HER modulation, T1-weighted structural MRI was performed for all subjects. Importantly, we applied Granger causality (GC) analysis^14^ to sources to identify information flows between the sources of HER modulations.

We formulated the following specific hypotheses. First, we expected that the HERs would be modulated by emotional expressions and that this would be revealed by different spatiotemporal dynamics between presentations of emotional and neutral stimuli. In particular, we used sad and happy expressions as emotional stimuli to observe an HER modulation effect after visual stimulus presentation because these emotional stimuli, as well as neutral faces, are known to be less influenced by the timing of the stimulus presentations within the cardiac cycle^15^. Although the emotional expression is not verified by this method, we expected that emoticons with these emotions (sad and happy) and neutral emoticons would also be less influenced by the timing of the stimulus presentations within the cardiac cycle. Second, we expected that modulation of the HERs by emotional expressions would be localized to the convergence regions of interoception and emotion, such as the anterior insula and anterior cingulate cortex^16^. Third, we expected that the information flows between these interoceptive regions would be modulated by emotional expression. More precisely, we expected that the bottom-up heartbeat information processing starting from the anterior insula, which represents the viscerosensory information from the posterior insula, to the anterior cingulate cortex would be enhanced by emotional expressions^12^. This pathway is hypothesized to be involved in the processing of the subjective salience of emotions using interoceptive signals^12,17,18^.

## Results

### Sensor analysis

**Figure 1:**
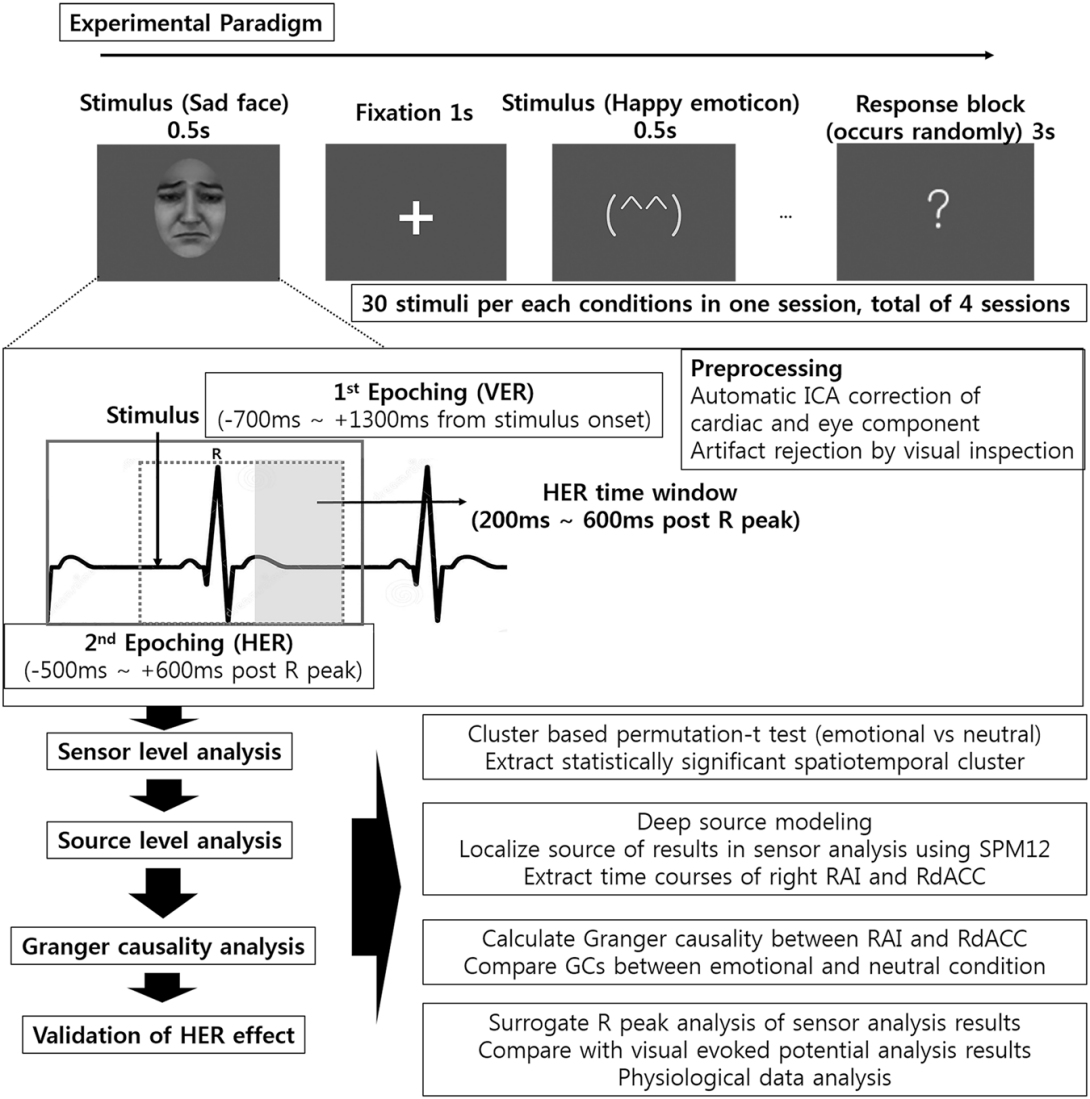
Experimental paradigm and overall analysis flow for MEG. Pictures of six conditions consisting of happy, sad, neutral faces and emoticons were presented to participants during MEG recording. Examples of emoticons are provided in Supplementary Fig. 1. Preprocessing including an automatic ICA correction of CFA and eye components using the Human Connectome Project MEG preprocessing pipeline were applied to a raw data. Examples of automatically rejected CFA-related components are provided in Supplementary Fig. 2. Then, HERs for each condition were extracted and cluster-based permutation *t* tests were performed to compare the HERs of the emotional and neutral conditions. In the source analysis, the sources of HER modulation found in the sensor analysis were localized. Among the sources of the HER modulation, a Granger causality analysis of the HER time courses between the right anterior insula (RAI) and right dorsal anterior cingulate cortex (RdACC) was performed to determine the change in the effective connectivity from an emotional condition that showed significant HER modulation in the previous steps. Finally, the HER modulation effect was validated using the surrogate R-peak analysis, visual-evoked response (VER) analysis, and physiological data analysis. The sad face used in this figure is different from the one that was used in the experiment (the sad face in this figure was generated by FaceGen Modeller (http://www.facegen.com/)).

Six conditions of stimuli consisting of happy face, sad face, neutral face, happy emoticon, sad emoticon, neutral emoticon were presented to forty healthy participants while recording magnetoencephalography (MEG) (Fig. 1). The stimuli were presented 120 times for each condition. Eight participants whose data had a magnetic field instability or abnormal ECG recording were excluded from further analyses. The continuous MEG data was filtered with a 1–30 Hz Butterworth filter. Eye movement and cardiac field artefacts (CFAs) were removed by independent component analysis (ICA) on the filtered continuous MEG data^19^ by the artefact correction algorithm used in the Human Connectome Project (HCP) MEG preprocessing pipeline^20,21^ (Fig. 1). Because a strong electromagnetic field produced by a cardiac activity could influence HERs, it is important to correct the CFAs^8,22^. After the epoching of the HERs of each condition, we compared the HERs of an emotional condition and a neutral condition. Four tests were performed, including a sad face vs. a neutral face, a happy face vs. a neutral face, a sad emoticon vs. a neutral emoticon, and a happy emoticon vs. a neutral emoticon. To address multiple comparison problems, we used cluster-based permutation paired *t* tests (Fig. 1).

### Significant difference in HERs between sad face and neutral face perceptions in the sensor analysis of HERs

**Figure 2:**
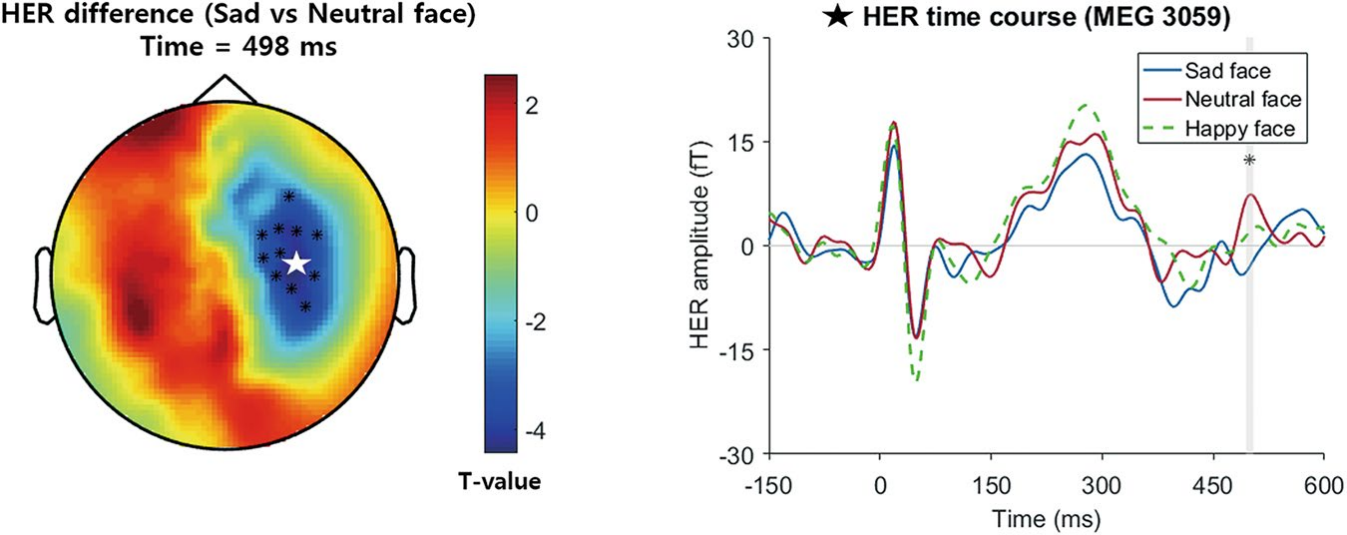
Topographic map (left) of differences in HERs between sad and neutral face conditions. Clusters showing significant differences are indicated by black dots (Monte-Carlo p = 0.044, cluster-corrected). In a single-channel plot of a significant cluster (right), the shaded area represents the cluster time window that showed a significantly different time course between conditions. The channel plotted in the right figure is indicated by a white star in the left topographic map.

A HER cluster showing a significant difference between the perceptions of a sad face and a neutral face was found in the right frontocentral sensors within the 494 ms–504 ms time range (Monte-Carlo p = 0.044, Fig. 2). No clusters were formed in other conditions, including happy face vs. neutral face, sad emoticon vs. neutral emoticon and happy emoticon vs. neutral emoticon.

### Source analysis

#### Interoceptive and prefrontal regions are the sources of HER modulations to sad faces

**Figure 3:**
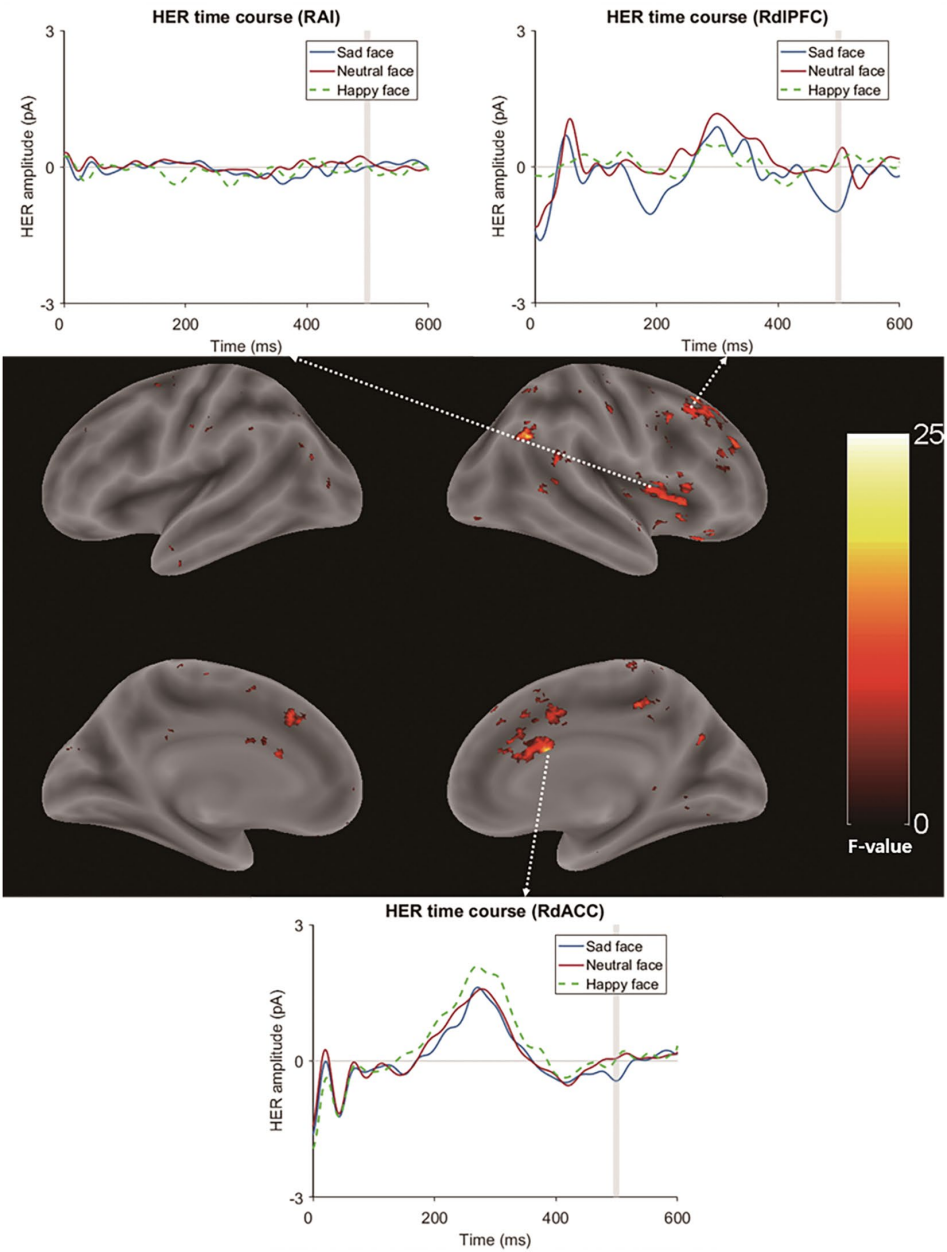
Brain regions showing significant differences in HER modulations in the contrast of sad vs. neutral face conditions for the mean time courses of HERs from the 25 voxels surrounding the peak voxels. RdACC, RAI, RdlPFC and basal ganglia clusters were found. The white dashed arrow connects the cluster region and its corresponding time course plot. The time windows used in the statistical test of the HER source analysis were shaded in the time course plot.

Source reconstruction was conducted using the MATLAB package Brainstorm^23^. To estimate the time courses of both the cortical and subcortical activities, we used the default settings in the open-source MATLAB toolbox Brainstorm’s implementation of the deep brain activity model using the minimum norm estimate (MNE)^23,24^ (Fig. 1). The source map was averaged over a time window of 494 ms to 504 ms, showing a significant difference between a sad face and a neutral face at the sensor level (other emotional conditions were not significantly different from neutral conditions in cluster-based permutation paired *t* tests). Then, this averaged spatial map was exported to SPM12 software^25^, and further statistical tests were performed (Fig. 1). Paired *t* tests were used to identify regions that had a different HER time course within the selected time window between sad and neutral faces. With the cluster-forming criteria of p-value < 0.01 and 10 adjacent voxels, several clusters of regions that had different HER time courses between sad face and neutral face conditions were identified. Six significant clusters appeared (Table 1). Briefly, the first cluster included the right dorsal anterior cingulate cortex (Table 1, Fig. 3, p < 0.001, cluster-level false discovery rate (FDR)-corrected). The second cluster included right dorsolateral prefrontal regions that consisted of the right superior frontal sulcus and the right middle frontal gyrus (Table 1, Fig. 3, p < 0.001, cluster-level FDR-corrected). The third cluster included the RAI (Table 1, Fig. 3, p = 0.001, cluster-level FDR-corrected). Note that previous studies have reported the ACC and AI as sources of HERs^10,26,27^. Other clusters were a basal ganglia cluster that included the right globus pallidus/right putamen (RGP/RP) cluster (Table 1, Fig. 3, p = 0.016, cluster-level FDR-corrected), another RdACC cluster (Table 1, Fig. 3, p = 0.042, cluster-level FDR-corrected), and another right dorsolateral prefrontal cortex (RdlPFC) cluster (Table 1, Fig. 3, p = 0.042, cluster-level FDR-corrected).

**Table 1:** Clusters (and their peak voxels) showing significantly different time courses between sad face and neutral face conditions in the HER analysis.

Cluster	p-value		No. of voxels	Region name (Destrieux atlas)	MNI coordinates (x, y, z)	F-value
	FWE-corrected	FDR-corrected				
Cluster 1 RdACC	<0.001	<0.001	391	G&S Cingul-Ant_R	7, 28, 22	19.42
				G&S Cingul-Mid_Ant_R	11, 24, 20	18.63
				S_Pericallosal_R	5, 10, 22	23.44
				G&S Cingul-Mid_Ant_L	−3, 22, 28	13.59
Cluster 2 RdlPFC	<0.001	<0.001	605	S_Front_Sup_R	21, 26, 38	15.38
				G_Front_Middle_R	35, 22, 42	11.04
				S_Front_Inf_R	33, 18, 36	12.57
Cluster 3 RAI	0.002	0.001	324	S_Circular_Insula_Sup_R	29, 24, 8	12.87
				G_Insular_Short R	33, 22, 6	12.43
				Putamen_R	31, 8, 8	12.87
Cluster 4 RGP/RP	0.074	0.016	186	Pallidum	23, −12, 0	12.14
				Putamen	25, −8, 0	12.17
Cluster 5 RdlPFC	0.266	0.042	140	G_Front_Middle_R	47, 42, 22	14.07
				G_Front_Inf_R	45, 42, 28	11.1
Cluster 6 RdACC	0.238	0.042	144	G&S Cingul-Mid-Ant_R	5, 12, 48	13.32
				G&S Cingul-Mid-Ant_L	−1, 10, 48	13.14
				G_Front_Sup_R	5, 16, 52	12.8

Voxel size = 1 × 1 × 1 mm; RdACC: right dorsal anterior cingulate cortex; RdlPFC: right dorsolateral prefrontal cortex; RAI: right anterior insula; RGP/RP: right basal ganglia; Cingul: cingulate; Front: frontal; G: gyrus; S: sulcus; Ant: anterior; Mid; middle; Sup: superior; R: right.

#### Increased GCs of HERs between RAI and RdACC induced by sad faces

**Figure 4:**
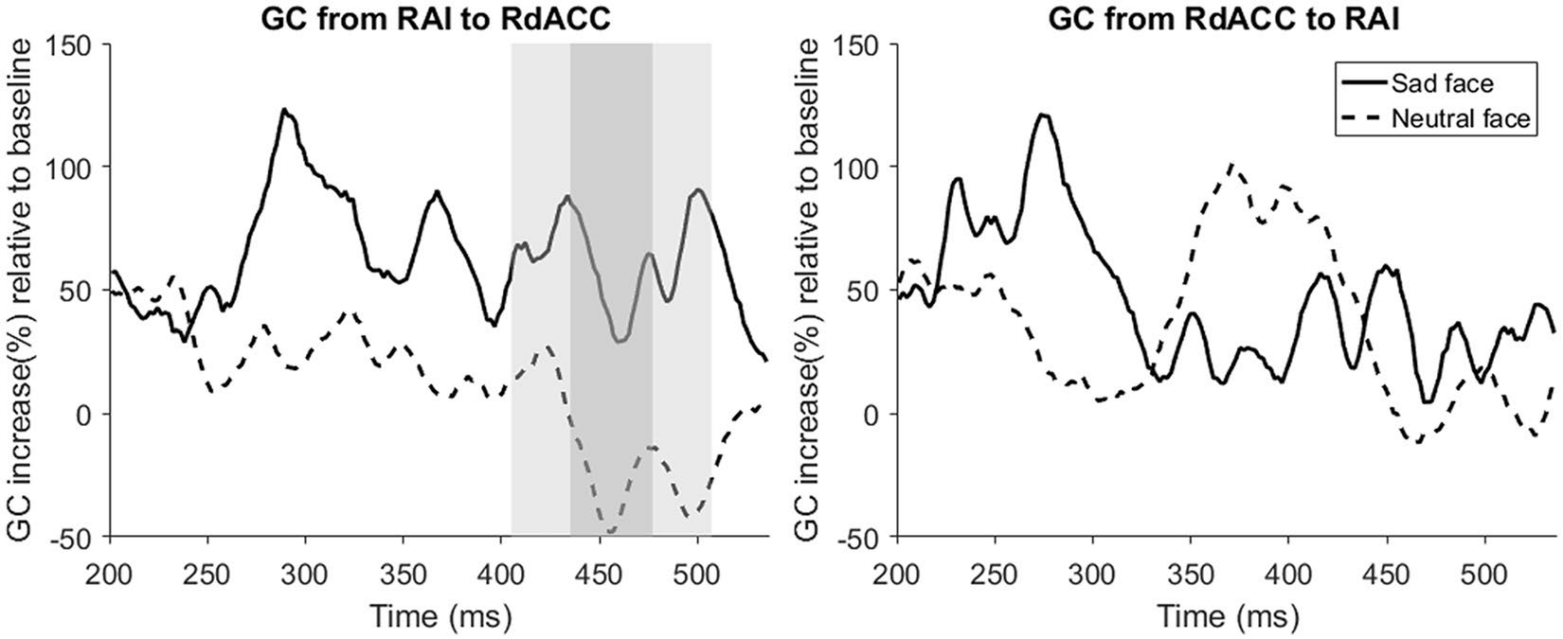
GC analysis results. There was an increased bottom-up transfer of interoceptive information from the RAI to the RdACC during sad face perception (left). The time in the figure represents the centre time of the sliding time windows post-R peak, and the shaded areas represent the time windows that showed significant differences between conditions after cluster corrections (Monte-Carlo p = 0.027, cluster-corrected; the densely shaded areas represent the centre time points of significant time windows, and the lightly shaded areas include the starting time point and the end time of the significant time windows).

After identifying brain regions that had different time courses, we performed a GC analysis^14^ on two regions of interest, the right anterior insula (RAI) and the right dorsal anterior cingulate cortex (RdACC), to determine whether the effective connectivity between these regions is modulated differently by sad faces compared to neutral faces (Fig. 1). These regions are known to be core regions of interoceptive processing and feelings^16,17,28^. Moreover, emotional processing in the AI and ACC is known to be right-lateralized, especially in the AI^11,29^. Short time window GC estimation with a sliding window was used, which is an appropriate method for an analysis whose temporal precision is important, such as an HER modulation effect. The pairwise GC was calculated between the RAI and the RACC from the (170 ms 230 ms) window to the (507 ms 567 ms) window. Then, we compared GCs of the sad face and neutral face conditions for all time windows using a cluster-based permutation paired *t* test. The time courses of these two regions were extracted from peak voxels of clusters containing RAI and RdACC, which were found in the source analysis of the HER. A pairwise GC analysis showed that only the GC of the HERs from the RAI to the RdACC was significantly higher in the sad face condition than in the neutral face condition between 406 ms and 507 ms (Fig. 4 (406 ms 466 ms) window to (447 ms 507 ms) window (shaded area), Monte-Carlo p = 0.027, cluster-corrected for 2 GCs and 174 time windows). The GC from the RdACC to the RAI was not significantly different between sad and neutral faces (no cluster was formed). These results indicate that only the bottom-up information from the RAI to the RdACC is increased.

### Control analysis

#### ECG time course difference between sad face and neutral face

To exclude the possibility that our HER modulation effect by sad faces was an artefact of the different electrical activities of the heart, we performed cluster-based permutation between ECG time courses of sad and neutral faces in the same way that we used in the sensor analysis (Fig. 1). There was no difference in the ECG time courses between sad and neutral faces, although they showed significantly different HER time courses (Supplementary Fig. 3a; no clusters were formed in the cluster-based permutation *t* test).

#### Heartbeat distributions in sad and neutral face conditions

**Figure 5:**
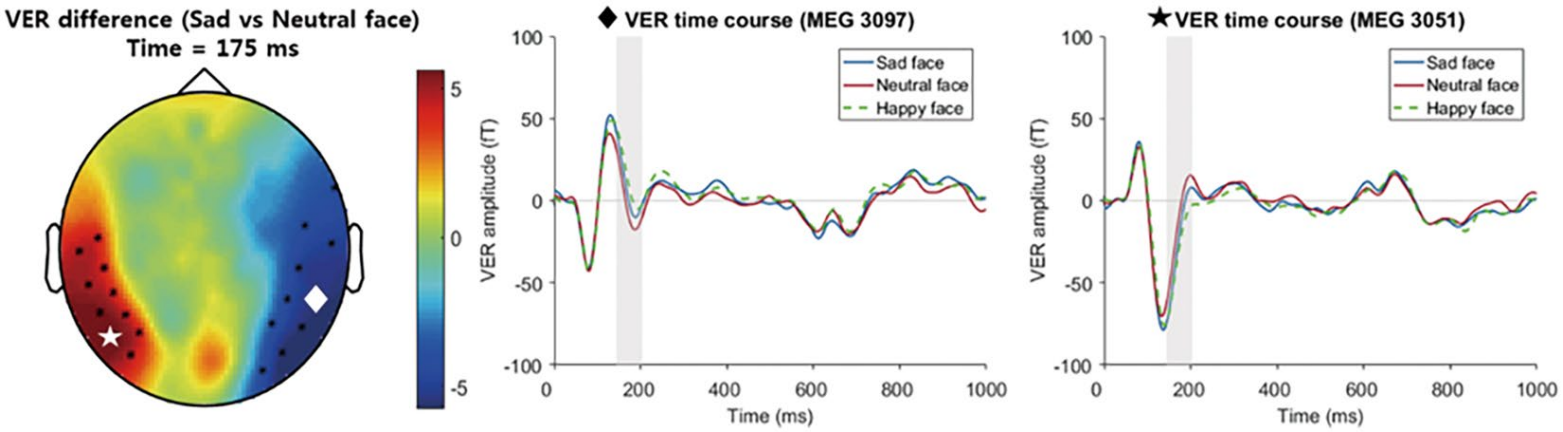
Topographic map (left figure) of differences in VERs between sad and neutral face conditions. Clusters showing significant differences between sad and neutral faces are indicated by black dots (left positive (red) cluster - Monte-Carlo p = 0.017, cluster-corrected, right negative (blue) cluster - Monte-Carlo p = 0.001, cluster-corrected). Representative time courses of the first cluster (negative cluster, 144 ms~204 ms post-stimulus, indicated by the white diamond in the topographic plot) and the second cluster (positive cluster, 151 ms~183 ms, indicated by the white star in the topographic plot) are plotted. The shaded areas represent the time points of clusters that showed significantly different time courses between conditions. The channels plotted in the middle and right figures are indicated by a white diamond and white star in the topographic maps, respectively.

To show that the HER modulation effect by sad faces was not the result of a biased heartbeat distribution, we divided the original visual epoch between −200 ms and 700 ms, i.e., the beginning and end of the HER epoching, into 100-ms time windows (a total of nine time bins) and counted the number of heartbeats in these time windows (Fig. 1). We completed this procedure for the sad and neutral face conditions. Then, the 2*9 two-way repeated-measures ANOVA of two conditions (sad and neutral faces) * nine time bins was performed to test whether the occurrence of the heartbeats was the same for both conditions and for every time bin. The results showed that there were no differences in the occurrence rate of heartbeats between the sad and neutral face conditions (F (1, 31) = 0.026, p = 0. 874, Supplementary Fig. 3b) and no differences in the occurrence rate of heartbeats between time bins (F (8, 248) = 1.435, p = 0.182, Fig. 5b).

#### Surrogate R-peak analysis of the HER modulation effect of a sad face

To test whether the HER modulation effect was time-locked to the heartbeat, we created 100 surrogate R peaks that were independent of the original heartbeats^26,30^ (Fig. 1). The surrogate R peaks were created by randomly shifting the original R peaks (−500 ms ~ +500 ms) by the same amount in each subject^30^. Then, we computed the surrogate HERs with the surrogate R peaks and performed the same cluster-based permutation *t* tests between the conditions that showed a significant difference in the sensor level analyses. Finally, we calculated the distribution of the maximum cluster statistics of the surrogate R peaks and the calculated position of our original cluster statistics in this distribution to show that the heartbeat-locked effect was significantly larger in such a distribution. The surrogate R-peak analysis showed that the size of the HER modulation effect (maximum cluster t statistics) was significant in the maximum cluster t statistics distribution of the surrogate R peaks (Monte-Carlo p < 0.03), indicating that our effect was highly likely to be time-locked to the heartbeat.

#### Different spatial patterns between visual-evoked response (VER) and HER analyses of sad face perceptions

**Figure 6:**
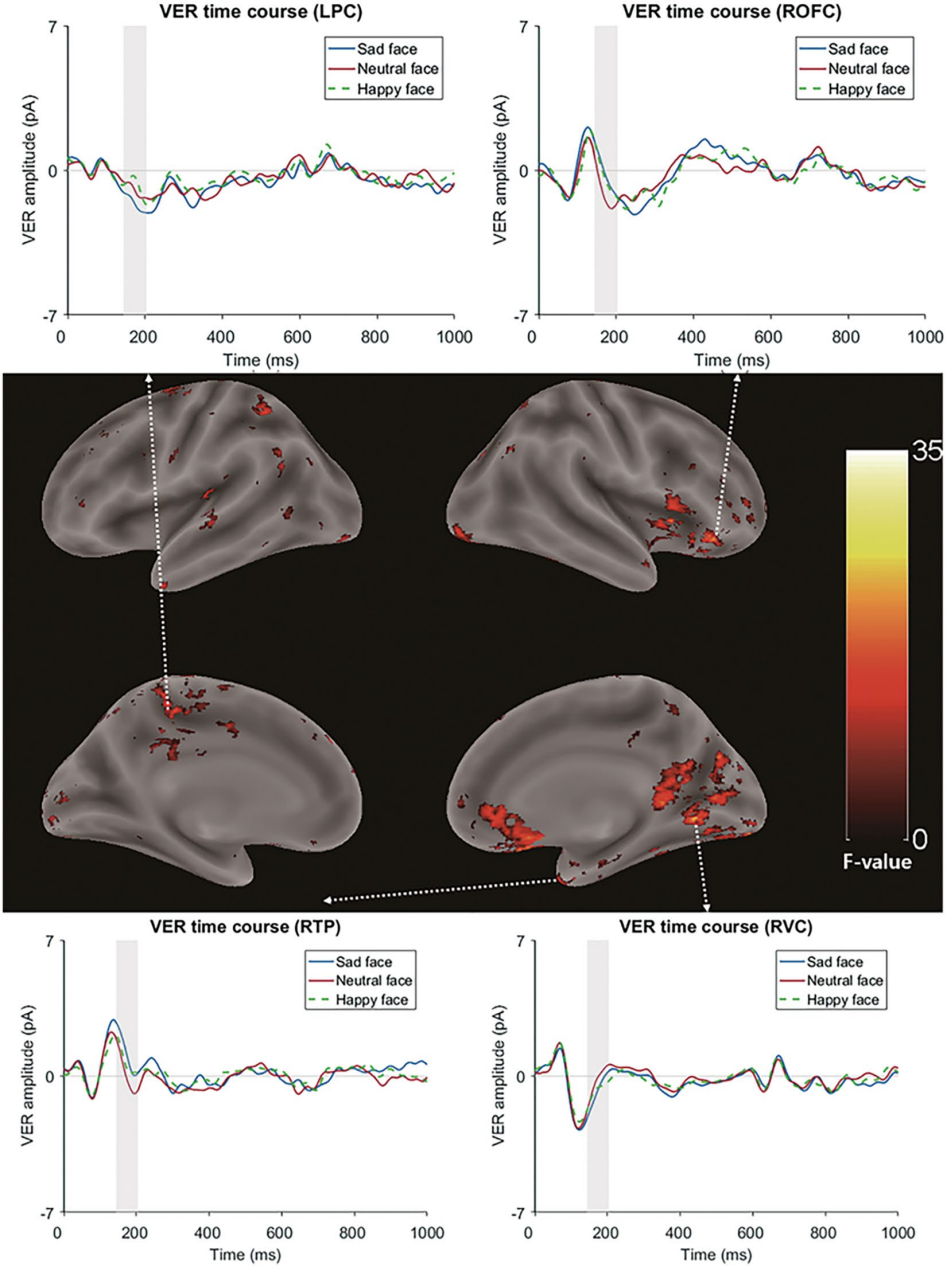
Regions showing significantly different VERs for a sad face compared to a neutral face (p < 0.01, cluster-level FDR-corrected, 144 ms~204 ms) and the mean time courses of VERs from the 25 voxels surrounding the peak voxels. Regions including the ROFC, RVC, and LPC/LPCC were found. The white dashed arrow connects the cluster region and its corresponding time course plot. The time windows used in the statistical test of the VER source analysis were shaded in the time course plots.

To test whether the HER modulation effect was similar to a VER effect, we performed the same cluster-based permutation test with visual stimulus-locked responses (Fig. 1). A time window of 0 ms to 1000 ms after the stimulus onset was used as the test input. We compared the topology of the significant clusters between HERs and VERs at the sensor level. Then, we performed source localizations of the VER activity in a significant cluster time window (144–204 ms) and exported them to SPM12 to perform statistical tests between emotional and neutral conditions with the same methods as used in the HER analysis. Next, we compared the resulting sources with the results of the HER analysis. This VER analysis was not performed for all conditions but instead was performed for the conditions that had a significant HER modulation effect so that we could compare the significant modulation of the HERs with the VER effect. In the cluster-based permutation *t* tests of VERs comparing sad vs. neutral face conditions, two significant clusters were found at 144 ms–204 ms (Monte-Carlo p = 0.001) and 151 ms-183 ms (Monte-Carlo p = 0.017) after the stimulus (Fig. 5). However, their topological distributions were completely different from those of the HER effect, which had a posterior-temporal distribution of significant clusters. In the source analysis of VERs (which was performed using the same method as the HER source analysis), six clusters were found at the threshold p < 0.01 with 10 adjacent voxels. In the first cluster, which was the largest cluster, most of the peak voxels were included in the right orbitofrontal cortices (ROFC, p < 0.001, cluster-level FDR-corrected, Table 2). This cluster also extended to the RAI and the right inferior frontal cortex (RIFC, Fig. 6), which was close to the RAI cluster found in the source analysis of the HERs (Supplementary Fig. 4). The second-largest cluster included regions of the right visual cortices (Table 2, Fig. 6). Other clusters were a left praecuneus/posterior cingulate cortex (LPC/LPCC) cluster, right temporal pole (RTP) cluster, left paracentral area cluster, and left precentral area cluster (Table 2, Fig. 6). Additionally, we tested whether there was overlap between the sources of HERs and VERs. We found that two voxels (1 × 1 × 1 mm in size) in the RAI overlapped with the source localization result of the HERs. Detailed information about the VER source analysis, including a list of peak voxels within each cluster, is provided in Table 2.

**Table 2:** Clusters (and their peak voxels) showing significantly different time courses between sad face and neutral face conditions in the VER analysis.

Cluster	p-value		No. of voxels	Region name (Destrieux atlas)	MNI coordinates (x, y, z)	F-value
	FWE-corrected	FDR-corrected				
Cluster 1 ROFC	<0.001	<0.001	6273	G_orbital_R	47 24 −21	31.32
				G_front_inf-Orbital_R	33 12 2	21.86
				S_orbital_med-olfact_R	11 26 −21	21.36
				G_and_S_cingul-Ant_R	9 26 −10	27.5
				G_subcallosal_R	6 14 −15	24.04
				G_oc_temp_med_Parahip_R	27 4 −13	23.35
Cluster 2 RVC	<0.001	<0.001	2074	G_oc-temp_med-Lingual_R	13 −60 −2	25.76
				S_oc-temp_med-Lingual_R	23 −75 −4	19.27
				G_oc-temp_lat-fusifor_R	23 −85 −13	22.86
				Pole_occipital_R	17 −87 −8	26.29
				Pole_occipital_L	−9 −101 −11	20.11
				S_calcarine_R	13 −56 12	22.16
				S_oc_sup_and_transversal_R	29 −75 24	25.14
				S_collat_transv_post_R	27 −83 −11	20.33
				Cerebellum_R	5 −62 0	23.09
Cluster 3 LPC/LPCC	0.006	0.002	280	S_cingul_Marginalis_L	−15 −42 56	21.56
				G_and_S_cingul_Mid_Post_L	−21 −28 44	14.23
				S_subparietal_L	−15 −40 46	15.55
				S_postcentral_L	−19 −40 48	17.74
Cluster 4 RTP	0.023	0.004	226	Pole_temporal_R	33 14 −43	34.26
Cluster 5 Left paracentral area	0.012	0.012	251	G&S_paracentral_L	−11 −46 76	18.98
				S_central_L	−13 −34 66	12.33
				S_cingul_Marginalis_L	−13 −34 60	11.88
Cluster 6 Left precentral area	0.022	0.022	229	S_precentral-sup-part_L	−26 −10 60	18.71
				G_Precentral_L	−13 −14 60	9.92
				S_precentral-inf-part_L	−34 −6 64	8.41
				G_front_sup_L	−28 −6 64	12.97
				G&S_cingul_Mid_Post_L	−13 −10 58	10.81
Cluster 7 RPCC	0.28	0.038	137	G_cingul-post-dorsal_L	−5, −46, 36	21.86
				G_cingul-post-dorsal_R	3, −30, 30	14.24
				S_pericallosal_L	−7, −44, 30	12.84
				S_subparietal_R	3, −36, 40	9.8
				S_subparietal_L	−5, −50, 40	8.67
Cluster 8 LPPC	0.185	0.027	152	G_pariet_inf-Angular_L	−38, −50, 56	19.06
				S_intralpariet_and_P_trans_L	−38, −46, 54	17.9
				G_parietal_sup_L	−38, −52, 60	16.57
Cluster 9 LPC	0.132	0.022	164	G_precuneus_L	−3, −58, 54	13.58
				G_precuneus_R	1, −64, 54	13.46

Voxel size = 1 × 1 × 1 mm; ROFC: right orbitofrontal cortex; RVC: right visual cortex; LPC: left praecuneus; LPCC: left posterior cingulate cortex; RTP: right temporal pole; RPCC: right posterior cingulate cortex; LPPC: left posterior parietal cortex; Cingul: cingulate; Front: frontal; Oc: occipital; Temp: temporal; G: gyrus; S: sulcus; Ant: anterior; Mid; middle; Sup: superior; R: right; L: left.

## Discussion

Our findings provide the first evidence that the perception of a sad face modulates bottom-up interoceptive information processing from the RAI to the RdACC.

First, we showed that cortical heartbeat processing after the presentation of a sad face had significantly different spatiotemporal dynamics from that of a neutral face and that these differences were localized to the interoceptive network (AI, dACC), prefrontal areas (dlPFC) and basal ganglia (GP, putamen). Importantly, the results of the GC analysis of these regions showed that bottom-up heartbeat information processing from the RAI to the RdACC was increased in the sad face condition. Visual-locked activity was localized to the ROFC, RVC and LPC/LPCC. Interestingly, the source of the VERs also included the RAI and was located in the neighbourhood of the source of the HERs. Finally, a surrogate R-peak analysis provided evidence that the HER modulation was time-locked to the heartbeat, which means that our results were a consequence of the cortical heartbeat processing modulation. Additionally, an analysis of the physiological data and CFA removal using ICA ruled out the possibility of other physiological effects on the cortical signal.

Our results extend previous studies of interoception and emotion in several aspects.

In the sensor analysis result, a sad face modulated HERs in the right frontocentral sensors at 494~504 ms post-R peak. One study showed that emotional arousal modulated HERs^8^. That study showed HER modulation by emotional arousal in the left parietal clusters 305 to 360 ms after the R peak and in a right temporoparietal cluster 380 to 460 ms after the R peak^8^. Another interesting recent study showed that HERs were modulated even in infants by emotional stimuli including fear and angry facial expressions, while no significant clusters were found in happy facial expressions^9^. Consistent with this study, the sad face, which is also a negative facial expression, modulated HERs in our study, while the happy face did not. Therefore, as Maister *et al*. argued in their paper, HERs are likely to be more sensitive to negative emotional processing^9^. However, the spatiotemporal patterns of the HER modulation were slightly different, in that video clips of an angry or fearful facial expression increased the HERs only 150 to 300 ms after the R peak in a frontal cluster^9^. It is hard to directly determine a difference in the spatiotemporal pattern of the HER modulation between that study and our study; however, it is notable that our results also showed an HER modulation effect in adults by the less-arousing emotional stimuli of sad faces. In particular, the fear and anger used in Maister *et al*. are both high-arousal emotions, while sadness is a low-arousal emotion^31^. It is likely that the interoceptive processing of the two types of negative emotion (high arousal vs. low arousal) might be different. For example, a recent study reported that the perception of fearful expressions was influenced by the timing of the cardiac cycle, while the perception of a sad face stimulus was not affected^15^; this finding indicates that there are different interaction patterns of interoception and emotional processing between fearful and sad faces, which might cause different HER patterns. Future studies of HERs using both types of negative emotional stimuli, with high and low arousal, could clarify these differences.

In the source analysis of HER and VER modulation by sad face perception, we found that there was both cardiac information processing and visual information processing in the neighbourhood of the RAI. For cardiac information processing, brain regions that reflect HER modulations during sensor analysis were found in the RdACC and the RAI, which were previously reported as sources of HERs^10,26,27,32^. In particular, HERs in the RdACC and RAI were found to be modulated by negative emotional stimuli in a previous study^10^. These regions were also identified in a recent meta-analysis as overlapping regions of emotion, interoception and social cognition processing^16^. In particular, the RdACC, which is one of the most important regions in the central autonomic network that regulates autonomic functions, was found to be a source of HERs induced by sad faces but not a source of VERs^33^. This region is known to be involved in negative emotion and pain processing^34^ and emotional awareness^17^. From these previous studies, it is likely that the RdACC is specifically involved in the interoceptive processing of negative emotional stimuli. In contrast to the RdACC, sources of both HERs and VERs were found in the RAI, and they were close to each other. The anterior insula is known for multimodal integration processing^18^. It is suggested that the RAI integrates autonomic signals with conscious thought processing^18^. Consistent with these studies, our results suggest the possibility that the RAI is involved in the integration of visual emotional information processing and cardiac interoceptive processing. Moreover, a GC analysis revealed that the cardiac information flow from the RAI to the RdACC increased during sad face perception. The RAI and RdACC are known to act conjointly in many situations, including the production of subjective feelings^12^ and are the main components of the salience network (SN)^18^. The processing of (emotionally) salient stimuli is influenced by ascending interoceptive and visceromotor signals that converge in the dorsal posterior insula (dPIC) and the mid-insula^18^. The SN integrates these ascending signals to coordinate large-scale networks in the cortex, such as switching between the default mode network (DMN) and central executive network (CEN)^18^. Moreover, within the SN, there was proven by GC analysis using fMRI to be an information flow from the RAI to the RdACC, while the reverse directional connectivity was weak^35^. Considering these studies, our results of increased GCs of HERs between the RAI and RdACC might reflect increased negative emotion-related saliency processing induced by sad faces, which includes the integration of interoceptive information and visual information. However, whether this processing is specific to sad emotions or relevant to subjective feelings cannot be determined from our results. Further GC studies of HERs using other emotional stimuli would clarify this point.

Although this is quite speculative, our result could be interpreted within the interoceptive predictive coding framework^36,37^. In this framework, emotion can be understood as an interoceptive inference, which means that an agent infers the likely cause of an interoceptive signal under an emotional context by updating an internal generative model. This process involves an interoceptive prediction (IP, corresponds to an emotional context) and the interoceptive prediction error (IPE) induced by the difference between the IP and the interoceptive signal. Interestingly, this process is hypothesized to involve the SN, especially in a way that a path from the AI to ACC processes the IPE^36,38^. By linking our result to this theory, an increased GC from the RAI to RdACC might reflect an IPE signal induced by emotional stimuli. However, because our experimental paradigms are not optimized to test hypotheses based on interoceptive predictive coding, further studies using a task with an appropriate design^22^ or an experiment that could control the interoceptive input signal such as vagus nerve stimulation (VNS), which was mentioned in a recent study^39^, should be performed.

In the source analysis of VERs, ROFC and RVC were seen, which is consistent with previous EEG/MEG studies of sad faces^40,41^. Most of these regions did not overlap with the source of the HERs (with the exception of one partial overlap of 2 voxels within the RAI).

An unexpected finding in the present study was that HERs were also modulated in the basal ganglia and dlPFC. The GP is known to send inputs to the prefrontal cortex via the thalamus. This pathway is involved in initiating motor actions. In particular, the ventral pallidum (VP) is closely involved in regulating emotion and initiating motor actions in response to emotional stimuli^42^. Moreover, a case has been reported of a patient with damage to the GP, including the VP, who reported an inability to feel emotions^43^. Based on this evidence, we suggest that cardiac information is relayed to the GP (including the VP) and finally the dlPFC, which plays a role in initiating emotion-related behaviours, such as facial expressions or the generation of feelings triggered by cardiac information processing; this processing is consistent with the somatic marker hypothesis.

One notable finding is that the HER modulation effect existed only for sad faces but not sad emoticons. An emoticon perception is context-dependent. One study reported that the addition of emoticons to messages increased arousal^44^. However, whether an emoticon itself without context would increase arousal is not known. The perception of emoticons without context would be influenced by many factors, such as personal emotional experience, considering that it is not an innate stimulus such as facial expressions. Consistent with this explanation, the mean variance of emotionality scores for sad emoticons across subjects was much larger than the variance of emotionality scores for sad faces both in the standardization cohort and MEG subjects, which might have caused varying degrees of emotional processing (and interoceptive processing) across the same category of stimuli. Therefore, further research controlling for this variance and the influence of the context using negative emoticons is necessary.

Additionally, our results showing the HER modulation effect only in the sad face condition could be due to selective attention to or preparation for negative stimuli. For example, most of the subjects had a strategy to look for sad faces during the discrimination task. In this case, the HER modulation may be found in the dACC or dlPFC, which are known to be related to task preparation or attention processes^45,46^. However, additional GG analyses did not show any significant HER modulation between the RdlPFC and RdACC or between the RdlPFC and RAI (supplementary materials). Furthermore, there was no difference in the accuracy of the discrimination task between emotional conditions. Therefore, it is unlikely that the attention or task preparation influenced our HER modulation results. Note that both additional GC analyses could indicate whether there was a ‘general’ increase or ‘specific’ increase in the GC between the RAI and RdACC from a sad face.

To the best of our knowledge, this work is the first to demonstrate that the processing of sad faces induces increased bottom-up processing of the HERs from the RAI to the RdACC. Moreover, both interoceptive information processing and visual processing, which are reflected by the HERs and VERs, were induced by sad faces. Additionally, we found that cardiac signals were processed differently in the basal ganglia and dlPFC during sad face processing, which might reflect the initiation of emotion-related behaviours.

## Methods

### Participants

Forty healthy participants (21 males and 19 females, mean age of 24.03 ± 3.28 years) volunteered for this experiment. The expected effect sizes were not known in advance, so we chose a sample size of approximately 40 participants, which was approximately two times the samples sizes of previous MEG and EEG studies of HERs^5,26,47^.

MEG recordings consisting of 4 runs were completed in one visit, and high-resolution T1-weighted MRI scans were acquired at another visit. In this MRI session, all subjects underwent both functional MRI experiments, consisting of emotional discrimination (unpublished data) and/or decision tasks (unpublished data), and other structural MRI scans, such as diffusion tensor imaging (unpublished data). We failed to acquire MEG data for five of the forty subjects due to magnetic field instability. Another three subjects were excluded during the analysis because their ECG data were too noisy or absent. Therefore, thirty-two subjects were included in the further analysis.

A structured interview was conducted using the Korean version of the Diagnostic Interview for Genetic Studies^48^. None of the subjects had current neurological or psychological diseases. All participants provided written informed consent to participate in the experiment. The study was approved by the Korean Advanced Institute of Science and Technology Institutional Review Boards in accordance with the Declaration of Helsinki.

### Standardization of emotional stimuli

The stimuli consisted of forty-five emotional faces and forty-five text-based emotional emoticons. Forty-five faces expressing happy, sad, and neutral emotions were selected from the Korean Facial Expressions of Emotions (KOFEE) database^49^. Text-based happy and sad emoticons were searched for on the world-wide web. Then, we created scrambled emoticons that did not have configurable information and used these scrambled emoticons as neutral condition emoticons (Figs 1,1). Ninety emotional expressions, including faces and text-based emoticons, were standardized in independent samples consisting of forty-seven healthy volunteers (21 females and 26 males, mean age of 28.43 ± 4.31 years). These participants were asked to rate the intensity of the feeling they felt towards the emotional expressions of 90 stimuli (45 faces and 45 facial emoticons with happy, neutral, and sad emotions) on an 11-point Likert scale (−5 to +5) using the instruction “Rate the intensity of feeling you felt about this picture”. We compared the mean absolute values of the four emotional expressions and two neutral expressions, which we called ‘feeling intensity’ or ‘emotionality’^50^. A repeated-measures analysis of variance (repeated-measures ANOVA) with a 2 stimulus (face, emoticon) by 3 valence (happy, sad, neutral) design was performed on the means and variances of the emotionality score independently. In the repeated-measures ANOVA of the means, there was a significant main effect of the valence (F (1.744, 80.228) = 272.618, p < 0.001, Greenhouse-Geisser-corrected), while there were no differences between emoticons and faces (F (1, 46) = 0.011, p = 0.919) and no interaction between those two main effects (F (1.685, 77.488) = 0.285, p = 0.818, Greenhouse-Geisser-corrected). In addition, a post hoc *t* test revealed that there was no difference between the sad and happy conditions (p = 0.082), but there was a significant difference between the emotional and neutral conditions (p < 0.001 for both sad and happy compared with neutral). In the repeated-measures ANOVA of the variance, the variance for the emoticons was significantly larger than the variance for the faces (F (1, 46) = 16.108, p < 0.001), while there was no difference in the variance between emotions (F (1.268, 58.342) = 2.608, p = 0.079, Greenhouse-Geisser-corrected) and no significant interaction (F (1.347, 61.963) = 4.831, p = 0.066, Greenhouse-Geisser-corrected). The participants from the main experiments also performed the above rating procedure before the MEG recordings, and we performed the additional repeated-measures ANOVA on this rating data in a same way as above. In the repeated-measures ANOVA of the means, there was a significant main effect of the valence (F (2, 30) = 256.615, p < 0.001, no difference between happy and sad in the post hoc *t* test with p = 0.822), significant main effect of emoticons and faces (F (1, 31) = 0.038, p = 0.038) and no interaction (F (2, 30) = 2.363, p = 0.111). Note that the emotionality scores of the emoticons were larger than those of the faces in the MEG subjects (t (31) = 2.164, p = 0.038, in post hoc *t* test). In the repeated-measures ANOVA of the variances, there was a significant main effect of the valence (F (2, 30) = 28.447, p < 0.001), a significant main effect of emoticons and faces (F (1, 31) = 39.064, p < 0.001) and a significant interaction (F (2, 30) = 42.422, p < 0.001).

### MEG experimental task

During the MEG recording, ninety stimuli consisting of 45 faces and 45 text-based emoticons were presented in the centre of a screen using in-house software, the KRISS MEG Stimulator 4 (Fig. 1). The size, duration, and stimulus onset asynchrony (SOA) of all the stimuli were 27 × 18 cm, 500 ms and 1500 ms, respectively, and the order of the presentation of the stimuli was pseudo-randomized. Participants completed 4 runs, each containing 180 stimuli (30 sad faces, 30 happy faces, 30 neutral faces, 30 sad emoticons, 30 happy emoticons, and 30 neutral emoticons) and lasting 270 s. In addition, to maintain participants’ attention to the task, when a question mark appeared, the participants had to discriminate by pressing buttons whether the emotional face or emoticon presented just before had a sad or happy emotion. This question mark appeared only after emotional stimuli and randomly appeared on the screen every 9 to 15 trials (mean = 11.3). A total of 4.7% of the trials was response trials. The mean accuracy of the discrimination task was 93.07%, and no difference in the accuracy was found between face and emoticon or between sad and happy (in a 2 (emoticon or face) by 2 (sad or happy) repeated-measures ANOVA, there was no main effect of face or emoticon: F (1, 31) = 0.001, p = 0.978, no main effect of sad or happy: F (1, 31) = 0.001, p = 0.978 and no interaction effect: F (1, 31) = 0.036, p = 0.851).

### Acquisition

A 152-channel MEG system (KRISS MEG, Daejeon, Korea, 152 axial first-order double-relaxation oscillation superconducting quantum interference device (DROS) gradiometers) covering the whole head was used for MEG recordings in a magnetically shielded room for 60–90 min at a sampling rate of 1,024 Hz. The relative positions of the head and the MEG sensors were determined by attaching four small positioning coils to the head. The positions of the coils were recorded at intervals of 10–15 min by the MEG sensors to allow co-registration with individual anatomical MRI data. The maximum difference deviations between the head positions before and after the run were <2 mm, and the goodness of fit (GoF) was >95%. EEGs of eye and muscle artefacts were recorded simultaneously with the MEG recordings. During the MEG recordings, participants were seated with their heads leaned back in the MEG helmet. The translation between the MEG coordinate systems and each participant’s structural MRI was made using four head position coils placed on the scalp and fiducial landmarks^51^.

### Data preprocessing

Data were processed with the FieldTrip toolbox^52^. First, continuous MEG data were filtered with a 1–30 Hz Butterworth filter. Then, eye movement and CFAs were removed by ICA on filtered continuous MEG data^19^ by the artefact correction algorithm used in the Human Connectome Project (HCP) MEG preprocessing pipeline^20,21^. Briefly, the independent component (IC) decomposition was performed iteratively. For each iteration, the ICs were classified as ‘Brain’ or ‘Noise’ using six parameters. The first three parameters were the correlation between (1) the IC and the electrocardiogram (ECG)/electrooculogram (EOG) channel time courses, (2) the IC and the ECG/EOG channel power time series (PTC), and (3) the IC and the ECG/EOG power spectral density (PSD). Three additional parameters derived from both the spectral and temporal properties were added to aid in the classification of system or environmental noise^21^. Finally, the iteration that had the highest brain component subspace dimensionality and the lowest residual artefact contamination was selected^20^. The average number of CFA-related ICs that were removed was 2.23 (Figs 1 and 2). Note that although the ICA is the most commonly used and reliable method to suppress the effect of CFAs, it is hard to completely remove the CFAs. Therefore, while we believe that the ICA removed most of the CFAs, it is not certain that there were no residual CFAs in our final preprocessed data. Second, the IC-removed data were epoched from 700 ms before the stimulus onset to 1300 ms after the stimulus onset. Then, the HERs for each stimulus condition were extracted by subsequent epoching, which was time-locked to the R peak of every epoch (Fig. 1). The R peaks were detected using the Pan-Tompkins algorithm^53^, and the HERs of each condition were extracted by epoching from 500 ms before the R peak to 600 ms after the R peak in the epoch of each condition. The average interbeat interval (IBI) was calculated by measuring the mean time interval between two consecutive R peaks in the whole data. The average IBI of all subjects was 905 ± 104 ms (heartrate = 66.3 BPM), with a range of 773 ms to 1126 ms (heartrate = 58.3 ~77.6 BPM). We also verified whether there was an IBI that was shorter than 600 ms (which might induce R-peak occurrence within another HER epoch). In 25 of 32 subjects, there was no IBI that shorter than 600 ms, and only 0.2% of the IBIs were shorter than 600 ms. Because a heartbeat is known to enter the central nervous system (CNS) approximately 200 ms after the R peak by vagal afferent stimulation at the carotid body^54^, while a visual stimulus enters the CNS immediately through the retina, a heartbeat that occurs 200 ms before a visual stimulus onset stimulates the brain earlier than a visual stimulus onset. Therefore, we excluded R peaks that occurred 200 ms before a stimulus onset to include only heartbeat-evoked processing that occurred after a visual stimulus. Therefore, we assumed that this procedure excluded most of the cortical inputs of a heartbeat that occurred before the visual stimulus. However, we cannot completely exclude this possibility because it is possible that an earliest cardiac input can enter the CNS within 200 ms after an R peak. R peaks at 700 ms after the stimulus onset were also excluded because that HER epoch would contain the next visual stimulus onset. Finally, the single epochs were inspected visually, and epochs containing artefacts were removed. The mean number of HER epochs per condition after the HER extraction procedure was 113.82, and there was no significant difference in the number of epochs between conditions (one-way repeated-measures ANOVA, F (5, 155) = 0.9151, p = 0.47). Finally, baseline correction was performed using a pre-R-peak interval of 150 ms, and trials on the same condition for each subject were averaged.

### Sensor analysis: Cluster-based permutation paired *t* tests between each emotional condition and the neutral condition

We compared the HERs of an emotional condition and a neutral condition. Four tests were performed, including a sad face vs. a neutral face, a happy face vs. a neutral face, a sad emoticon vs. a neutral emoticon, and a happy emoticon vs. a neutral emoticon. To address multiple comparison problems, we used cluster-based permutation paired *t* tests. These tests were performed as follows. First, the data were downsampled to 512 Hz to make the computation efficient, and paired *t* tests were performed at every time point between 200 and 600 ms and all sensors. Then, the significant spatiotemporal points of uncorrected p-values below 0.005 (two-tailed) were clustered by the spatiotemporal distance, and the summed t-value of each cluster was calculated. After the calculation of the cluster t-stat, a permutation distribution was created by randomly switching condition labels within subjects, calculating the t-value of the paired *t* test between permutated conditions, forming clusters as mentioned above, selecting the maximum cluster t-value and repeating this procedure 2000 times. Finally, after the maximum cluster t-values of each permutation were used to create a permutation distribution, the corrected-p-value original clusters were calculated.

### Source analysis

Source reconstruction was conducted using the MATLAB package Brainstorm^23^. To estimate the time courses of both cortical and subcortical activities, we used the default settings in the open-source MATLAB toolbox Brainstorm’s implementation of the deep brain activity model using the MNE^23,24^. First, cortical surfaces and subcortical structures, including the amygdala and basal ganglia, were generated for each subject from 3T MPRAGE T1 images using FreeSurfer^55^. The individual heads/parcellations were then read into Brainstorm^23^ along with the tracked head points to refine the MRI registration. In Brainstorm, a mixed surface/volume model was generated, and 15,000 dipoles were generated on the cortical surface with another 15,000 dipoles generated in the subcortical structure volume. Refining the registration with the head points improves the initial MRI/MEG registration by fitting the head points digitized at the MEG acquisition and the scalp surface. Using the individual T1 images and transformation matrix generated as above, a forward model was computed for each subject using a realistic overlapping sphere model. The source activity for each subject was computed using the MNE (Brainstorm default parameters were used). The source map was averaged over a time window of 494 ms to 504 ms, and there was shown to be a significant difference between a sad face and a neutral face at the sensor level (other emotional conditions were not significantly different from the neutral conditions in cluster-based permutation paired *t* tests). Then, this averaged spatial map was exported to SPM12 software^25^, and more statistical tests were performed. Paired *t* tests were used to identify regions that had different HER time courses for sad and neutral faces within the selected time window.

### GC analysis of HER source activity

After identifying the brain regions that had different time courses, we performed a GC analysis^14^ on two regions of interest, the RAI and the RdACC, to determine whether the effective connectivity between these regions is modulated differently in emotional conditions compared to a neutral condition. These regions are known from many previous studies to be core regions of interoceptive processing and feelings^16,17,28^. Moreover, emotional processing in the AI and ACC is known to be right-lateralized, especially in the AI^11,29^. The time courses of these two regions were extracted from peak the voxels of clusters containing the RAI and RdACC, which were found in the source analysis of the HER. The coordinates of the RAI and the RdACC were (29, 24, 8) and (7, 28, 22), respectively, and included 25 adjacent voxels. In the GC analysis^56^, time course Y Granger-causes time course X if the k past time point data of both X $({X}_{t-k},\,{X}_{t-k+1,}\ldots {X}_{t-1})$ and Y $({Y}_{t-k},\,{Y}_{t-k+1,}\ldots {Y}_{t-1})$ predict X at time t (*X*_*t*_) better than the past time point data of X alone. Therefore, Granger causality is also called Granger prediction and measures the predictive causality. The GC is formulated by the log-likelihood ratio between the residual covariance matrix of the model that explains X by the pasts of X and Y and the residual covariance matrix of the model that explains X by the past of X alone^14^.

$${X}_{t}=\sum _{k=1}^{p}{A}_{xx,k}\,\ast \,{X}_{t-k}+\sum _{k=1}^{p}{A}_{xy,k}\,\ast \,{Y}_{t-k}+{{\rm{\varepsilon }}}_{x,t}$$

$${X}_{t}=\sum _{k=1}^{p}{A^{\prime} }_{xx,k}\,\ast \,{X}_{t-k}+{{\rm{\varepsilon }}^{\prime} }_{x,t}$$

$${{\rm{F}}}_{{\boldsymbol{Y}}\to {\boldsymbol{X}}}=\,\mathrm{ln}\,\frac{|{\rm{\Sigma }}^{\prime} {\rm{xx}}|}{|{\rm{\Sigma }}\mathrm{xx}|}$$

A is the matrix of the regression coefficient, epsilon is the residual, sigma is the covariance matrix of the residual, and F is the GC of X and Y. The first equation presents a regression model that predicts the time course of region X at time point t (*X*_*t*_) using previous time points of both X and another region Y, and the second equation represents the regression model that predicts *X*_*t*_ using only the previous time point of X. The GC(F_***Y***→***X***_) is formulated by the log-likelihood ratio between the residual covariance matrices $({\rm{\Sigma }}\mathrm{xx}={\rm{cov}}({{\rm{\varepsilon }}}_{x,t})$ and ${\rm{\Sigma }}x{\rm{x}}^{\prime} ={\rm{cov}}({{\rm{\varepsilon }}^{\prime} }_{x,t}))$. All calculations were performed using a multivariate GC toolbox (MVGC toolbox)^14^.

The time courses of two ROIs were extracted for every trial for each subject. To satisfy the stationarity assumption of the GC analysis, we used a short time window approach for GC estimations with sliding windows^57,58^. This approach was also appropriate for our analysis of the HER modulation effect, which was identified in a very short time window and thus required a high temporal precision for the GC estimation. The size of the window was 60 ms, the step size was 2 ms^57,59^, and the GC calculation was performed for the whole epoch, which started from the (−500 ms −440 ms) window to the (540 ms 600 ms) window. Stationarity was further controlled by removing the average event-related responses from each trial^60^. The model order was determined using the Akaike information criterion (AIC) to a maximum order of seven time points, which corresponded to 14 ms. After the model estimation, we tested the stationarity of the model by examining whether the spectral radius ρ(A) > 1 in every time window and every subject^14^. Although we tried to control every time window to satisfy the stationarity assumption, after the (507 ms 567 ms) time window and from the (−137 ms −77 ms) to the (77 ms 137 ms) window, there were participants who violated the stationarity assumption. A similar pattern was observed even when we tested variable lengths of the time window and model orders. We suspected that this stationarity violation might be induced by CFAs. Therefore, we used the time window starting from the (170 ms 230 ms) window to the (507 ms 567 ms) window, and every time window satisfied the stationarity assumption in every participant. Pairwise GC analyses of the two ROIs (two GCs: RAI to RdACC and RdACC to RAI) were performed for emotional and neutral conditions. To compare the emotional and neutral conditions, GC baseline normalization was performed in both conditions by calculating the change in the GC relative to the average GC between the (−257 ms −197 ms) window and the (−167 ms −107 ms) window such that difference between the end point and starting point of the baseline is equal to the length of the baseline that we used in the previous analyses (150 ms)^59^. Time windows approximately 0 ms post-R peak were not used as a baseline because three subjects violated the stationarity assumption. Finally, the 2 estimated GCs of emotional and neutral conditions were compared using a cluster-based permutation paired *t* test for all time windows starting from the (170 ms 230 ms) window to the (507 ms 567 ms) window^52^. Therefore, the multiple comparisons were controlled for the number of GCs and the number of time windows using a cluster-based permutation paired *t* test.

## Electronic supplementary material

Supplementary_Information

## Data Availability

The dataset in this study is available from the corresponding author upon reasonable request.
