# Supplementary material for: Sad faces increase the heartbeat-associated interoceptive information flow within the salience network: a MEG study
Source: Sci Rep. 2019 Jan 23;9:430. doi: 10.1038/s41598-018-36498-7 (PMC6344475; doi:10.1038/s41598-018-36498-7)
Supplement: Supplementary file 1 — Supplementary_Information [file 41598_2018_36498_MOESM1_ESM.pdf]

Supplementary information - Sad faces increase the heartbeat-associated interoceptive information flow within the salience network: a MEG study

Author Affiliations: Jaejoong Kim[1,5], Hyeong-Dong Park[2], Ko Woon Kim[1,3], Dong Woo Shin[1,5], Sanghyun Lim[4], Hyukchan Kwon[4], Min-Young Kim[4], Kiwoong Kim[4], Bumseok Jeong[1,5*]

[1]Graduate School of Medical Science and Engineering, Korea Advanced Institute for Science and Technology (KAIST), 291 Daehak-ro, Yuseong-gu, Daejeon, 34141, Republic of Korea; [2]Laboratory of Cognitive Neuroscience, Center for Neuroprosthetics and Brain Mind Institute, Ecole Polytechnique Fédérale de Lausanne (EPFL), 9 Chemin des Mines, 1202 Geneva, Switzerland; [3]Department of Neurology, Chonbuk National University Hospital, Chonbuk National University Medical School, JeonJu, Korea; [4]Center for Biosignals, Korea Research Institute of Standards and Science, Daejeon, South Korea; [5]KI for Health Science and Technology, KAIST Institute, KAIST, 291 Daehak-ro, Yuseong-gu, Daejeon, 34141, Republic of Korea

Correspondence: Bumseok Jeong, M.D., Ph.D., Associate Professor, Director, Laboratory of Computational Affective Neuroscience and Development, Graduate School of Medical Science and Engineering, Korea Advanced Institute of Science and Technology, Daehak-ro 291, Daejeon, Korea

Tel. +82-42-350-4245 (Office), **E-mail:** bs.jeong@kaist.ac.kr

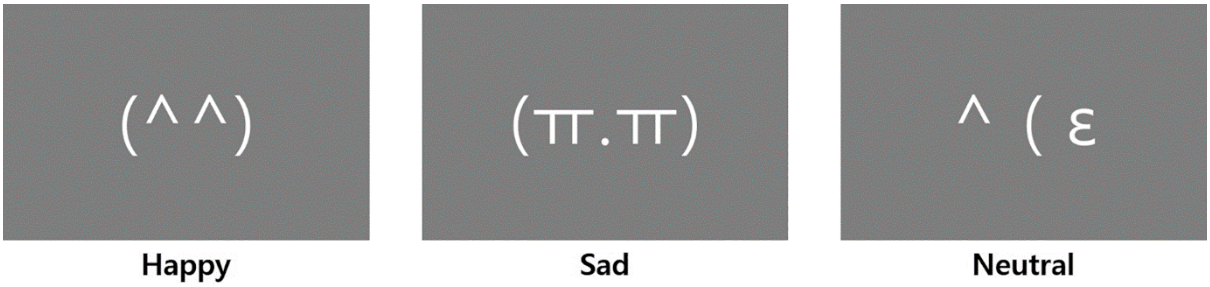

Supplementary figure 1. Examples of text-based emoticons.

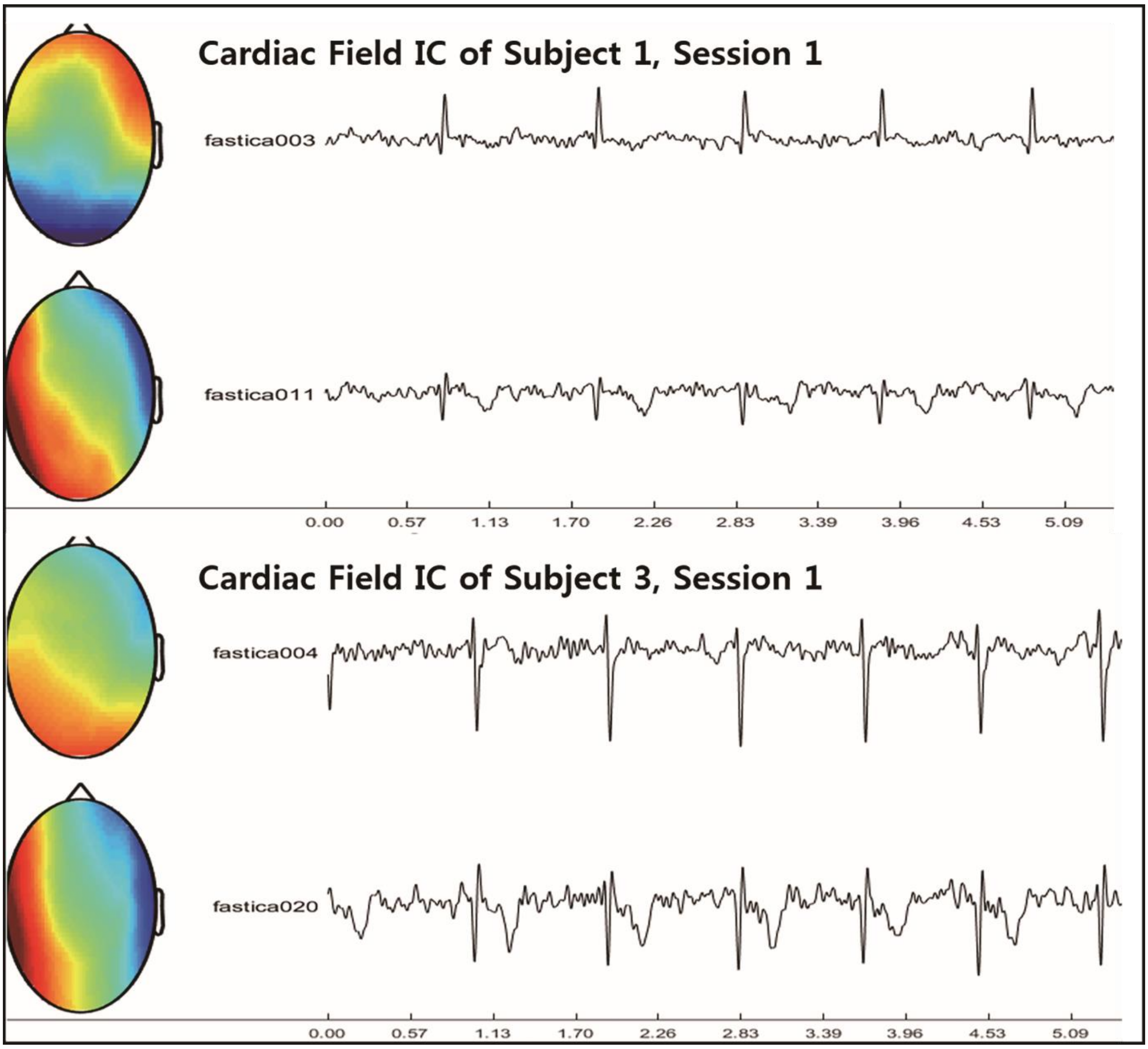

Supplementary figure 2. Examples of CFA-related ICs, which were automatically detected using HCP MEG preprocessing pipeline.

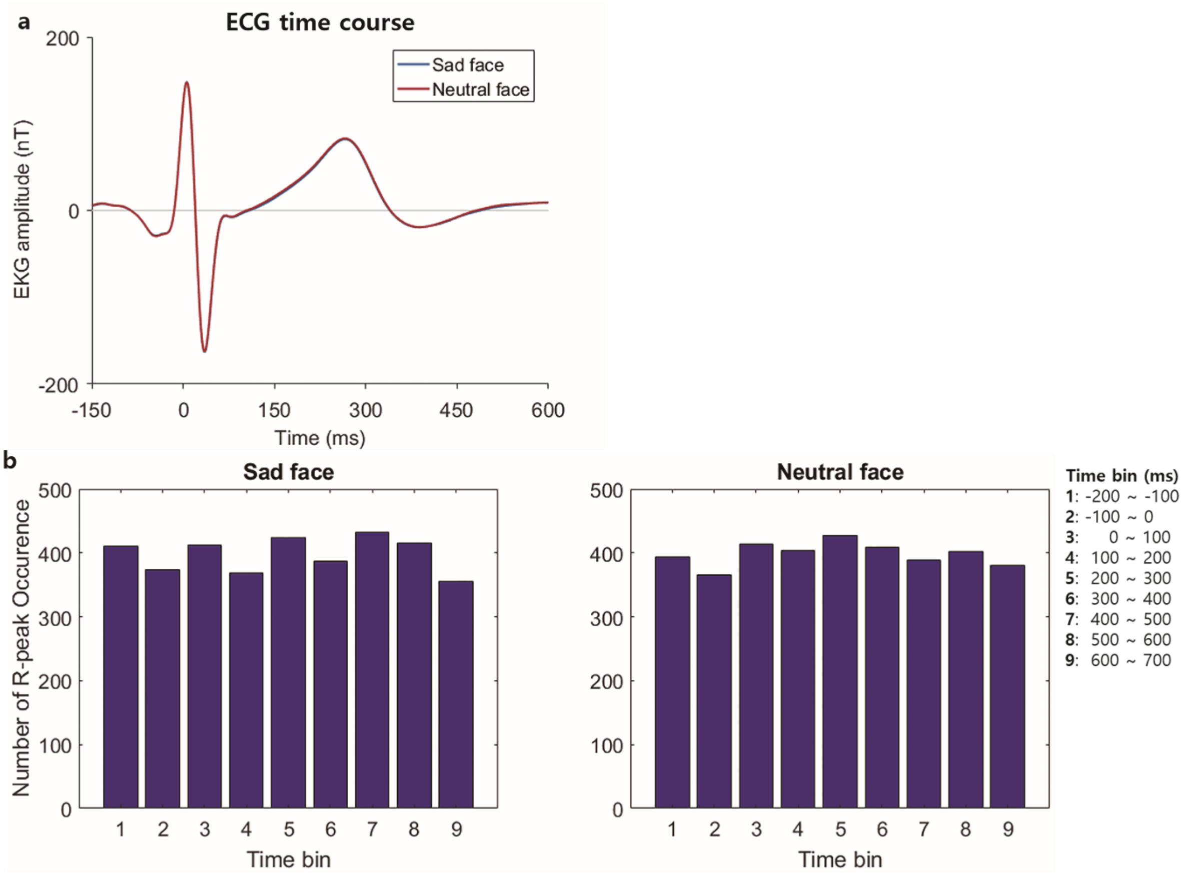

Supplementary figure 3. Physiological data analysis results. a. Average ECG time course of sad and neutral faces which showed no significant differences between conditions (no clusters were formed in cluster based permutation paired *t* test). b. Total R-peak occurrence distributions in epochs of sad face (left) and neutral face (right) conditions. There was no difference in the occurrence rate of heartbeats between conditions and also there was no difference of heartbeat occurrence between time bins (all $p > 0.1$).

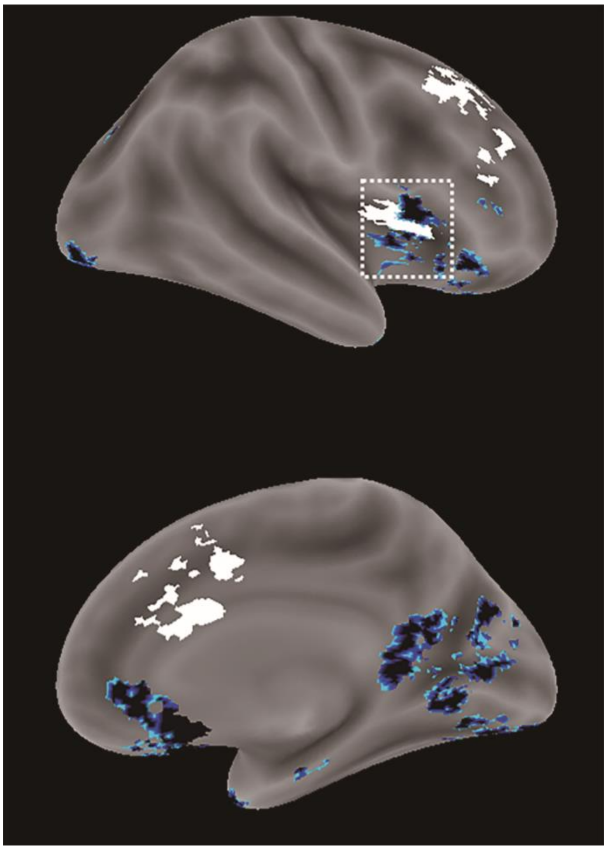

Supplementary figure 4. Neighbouring of localized sources of HER and VER modulation by sad face in the RAI (in white dotted square). The sources of HERs are coloured white, and the sources of VERs are coloured dark blue.

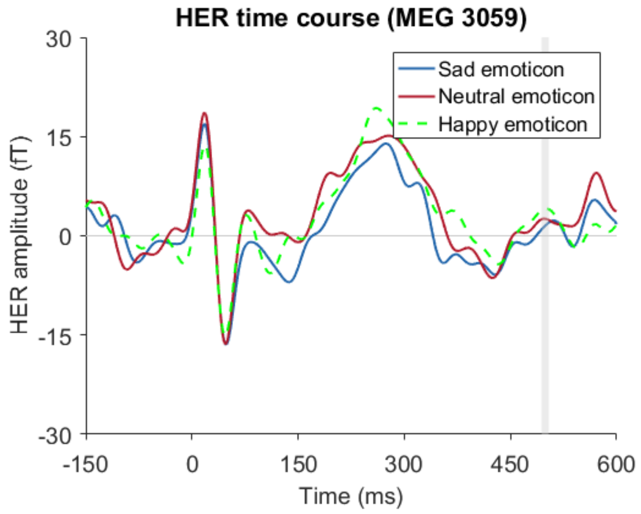

Supplementary Figure 5. Sensor time courses of the HER of emoticons

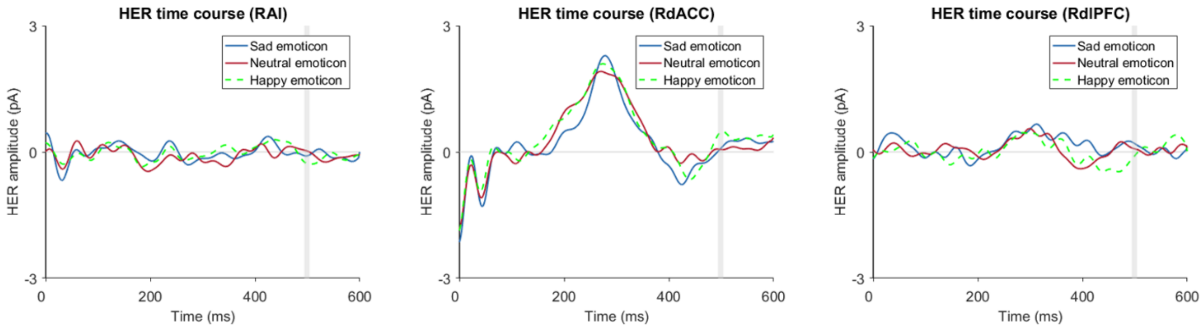

Supplementary Figure 6. Source time courses of the HER of emoticons.

Additional cluster based permutation paired *t* test between happy face condition and sad face condition

In order to verify whether the HER of sad faces were different only with HER of neutral faces or they were also different with the HER of happy faces (those have opposite valence with sad faces), we performed an additional cluster based permutation paired *t* test between happy face condition and sad face condition (by using the same method used in the previous sensor analysis). However, there was no significant difference between these conditions (no clusters were formed).

Additional GC analyses between RAI-RdlPFC and RdACC-RdlPFC

We performed 2 additional GC analyses – GC analysis between RAI and RdlPFC and GC analysis between RdACC and RdlPFC whose purpose is 1) to exclude the possibility that a preparation or attention (for upcoming discrimination task) related cortical processing influenced the HER processing which might have influenced our HER modulation results, especially in RdACC and RdlPFC whose interaction is known to be involved in preparation and sustaining attention to the task [1,2], and 2) to test the possibility whether there was a general increase of GCs induced by sad faces or the increase of GC was specific to RAI to RdACC. We estimated pairwise GCs between these regions and performed cluster based permutation *t* tests. The MNI coordinate of the RdlPFC used in these analyses was (21, 26, 38) which was the most significant in the RdlPFC cluster and 25 adjacent voxels were included. However, there were no significantly increased or decreased GCs in both additional GC analyses (Monte-Carlo $p = 0.4313$ in GC analysis between RAI and RdlPFC and Monte-Carlo $p = 0.4338$ in GC analysis between RdACC and RdlPFC). Therefore, we concluded that it is not likely that an increased GC from RAI to RdACC is resulted from an increased attention to a discrimination task or general increase of GCs induced by sad faces.

Source analysis using more stringent cluster defining threshold

Recent studies reported that a liberal cluster defining threshold (CDT) in neuroimaging studies could cause more false positive rate than expected [3,4] and recommended a stringent cluster defining threshold (CDT) of $p < 0.001$. Thus, we tested with cluster defining threshold (CDT) of both $p < 0.005$ and $p < 0.001$. The CDT $p < 0.005$ was used in recent research of MEG whose statistical tests were done using SPM [5]. No cluster was survived in the CDT $p < 0.001$. However, in the CDT $p < 0.005$, the RdlPFC cluster (cluster $P_{FDR} < 0.001$, cluster extent = 352), and the RAI/putamen cluster (cluster $P_{FDR} = 0.001$, cluster extent = 226) were survived while the dACC cluster was failed to survive (cluster $P_{FDR} = 0.065$, cluster extent = 80). Therefore, we verified that RAI and RdlPFC clusters were reliable under more stringent CDT of $p < 0.005$ while other clusters reported in our study including the dACC cluster were relatively less reliable than these clusters.

References

1 MacDonald, A. W., Cohen, J. D., Stenger, V. A. & Carter, C. S. Dissociating the role of the dorsolateral prefrontal and anterior cingulate cortex in cognitive control. *Science* **288**, 1835-1838 (2000).

2 Shenhav, A., Botvinick, M. M. & Cohen, J. D. The expected value of control: an integrative theory of anterior cingulate cortex function. *Neuron* **79**, 217-240 (2013).

3 Eklund, A., Nichols, T. E. & Knutsson, H. Cluster failure: why fMRI inferences for spatial extent have inflated false-positive rates. *Proceedings of the National Academy of Sciences*, 201602413 (2016).

4 Kessler, D., Angstadt, M. & Sripada, C. S. Reevaluating "cluster failure" in fMRI using nonparametric control of the false discovery rate. *Proceedings of the National Academy of Sciences* **114**, E3372-E3373 (2017).

5 Sanders, R. D., Winston, J. S., Barnes, G. R. & Rees, G. Magnetoencephalographic Correlates of Perceptual State During Auditory Bistability. *Scientific reports* **8**, 976 (2018).
